# Parameter Optimization Using Covariance Matrix Adaptation—Evolutionary Strategy (CMA-ES), an Approach to Investigate Differences in Channel Properties Between Neuron Subtypes

**DOI:** 10.3389/fninf.2018.00047

**Published:** 2018-07-31

**Authors:** Zbigniew Jȩdrzejewski-Szmek, Karina P. Abrahao, Joanna Jȩdrzejewska-Szmek, David M. Lovinger, Kim T. Blackwell

**Affiliations:** ^1^Krasnow Institute of Advanced Study, George Mason University, Fairfax, VA, United States; ^2^Laboratory for Integrative Neuroscience, Section on Synaptic Pharmacology, National Institute on Alcohol Abuse and Alcoholism, National Institutes of Health, Rockville, MD, United States; ^3^Department of Bioengineering, Volgenau School of Engineering, George Mason University, Fairfax, VA, United States

**Keywords:** striatum, globus pallidus, MOOSE, neuronal model, biophysics, ion channels

## Abstract

Computational models in neuroscience can be used to predict causal relationships between biological mechanisms in neurons and networks, such as the effect of blocking an ion channel or synaptic connection on neuron activity. Since developing a biophysically realistic, single neuron model is exceedingly difficult, software has been developed for automatically adjusting parameters of computational neuronal models. The ideal optimization software should work with commonly used neural simulation software; thus, we present software which works with models specified in declarative format for the MOOSE simulator. Experimental data can be specified using one of two different file formats. The fitness function is customizable as a weighted combination of feature differences. The optimization itself uses the covariance matrix adaptation-evolutionary strategy, because it is robust in the face of local fluctuations of the fitness function, and deals well with a high-dimensional and discontinuous fitness landscape. We demonstrate the versatility of the software by creating several model examples of each of four types of neurons (two subtypes of spiny projection neurons and two subtypes of globus pallidus neurons) by tuning to current clamp data. Optimizations reached convergence within 1,600–4,000 model evaluations (200–500 generations × population size of 8). Analysis of the parameters of the best fitting models revealed differences between neuron subtypes, which are consistent with prior experimental results. Overall our results suggest that this easy-to-use, automatic approach for finding neuron channel parameters may be applied to current clamp recordings from neurons exhibiting different biochemical markers to help characterize ionic differences between other neuron subtypes.

## Introduction

Computational models of neurons and networks are being used increasingly to test hypotheses regarding causation of biological mechanisms, e.g., ion channels, on neuron function. For example, the effect of blocking an ion channel on neuron activity (Tucker et al., 2012; Qian et al., 2014), the effect of a synaptic connection on network activity (Prinz et al., 2004; Damodaran et al., 2015), or the effect of morphology on neuron firing patterns (Schaefer et al., 2003; Meza et al., 2018) can be tested by computational models. Unlike in wet lab experiments, neither non-specific effects of drugs nor compensatory effects during development confound the results. Computational models also can be used to determine whether the observed differences in voltage trajectory (e.g., action potential width or firing rate) between neuron classes correspond to differences in ion channel conductances (Rumbell et al., 2016).

Whereas the simulation experiments (comparison of control and treatment models) are relatively simple, creation of the control model is exceedingly difficult. Developing a biophysically realistic, single neuron model requires equations describing ionic channel kinetics developed from voltage clamp data (Gurkiewicz and Korngreen, 2007; Taylor et al., 2009), cell morphology (Segev and London, 2000; Van Ooyen et al., 2002), and then ionic channel conductances are adjusted to match firing properties of the target neuron. The number of parameters and the non-linear interactions between ionic channels makes adjusting the parameters an extremely difficult problem. Furthermore, changes in current density of an outward current can be compensated by similar changes to inward current density or opposite changes to other outward currents (Marder and Goaillard, 2006). Thus, a single neuron class has numerous sets of parameters that produce the same observed physiology.

Several approaches have been developed recently for automatically adjusting parameters of computational neuronal models. Given the increase in computing power, the number of publications is increasing; thus, for brevity, we will mostly discuss the recent publications and refer the reader to a previous review of earlier publications (Van Geit et al., 2008). These methods vary not only in the search technique (i.e., the method of sampling the parameter space), but also in the fitness function used and the data used to fit the model. Perhaps the most successful approach is to fit a model to simulated data (Vanier and Bower, 1999; Brookings et al., 2014). The advantage of this approach is that a known solution exists. The disadvantage is that the goal of most parameter optimization is to fit electrophysiology data, which is a more difficult undertaking.

All optimization algorithms use one or more fitness functions (also called cost functions), which are measures of similarity between the model and the experiment. Comparing simulated and experimental voltage traces directly is a difficult problem, because a millisecond change in spike time, which misaligns the spikes, may produce a large difference in Euclidean distance between traces (though only a minor change in perceived similarity). A clever solution to this problem is to apply an adjustment in the simulation values, based on the difference between experimental and simulated values, to promote alignment of the traces (Abarbanel et al., 2009; Brookings et al., 2014). If multiple data traces are being fit, the similarity of each trace needs to be weighted to calculate an overall similarity value. A more common solution is to extract features of the voltage traces, such as spike width and firing rate, and then either combine them into a single objective (Holmes et al., 2006; Rumbell et al., 2016) or use a multi-objective optimization method (Druckmann et al., 2007; Hay et al., 2011; Rumbell et al., 2016; Neymotin et al., 2017). Feature extraction avoids the problem of spike alignment, but compounds the problem of how to weight the different features when combined into a single-objective.

Most of the modern search methods use variants of evolutionary algorithms (Vanier and Bower, 1999; Keren et al., 2005; Hendrickson et al., 2011b; Brookings et al., 2014; Martínez-Álvarez et al., 2016; Rumbell et al., 2016; Martínez-Cañada et al., 2017; Neymotin et al., 2017). The covariance matrix adaptation evolutionary strategy is a modern evolutionary algorithm that works quite well for large numbers of parameters (Hansen and Kern, 2004). CMA-ES combines an evolutionary approach with a model of the fitness landscape. In an evoluationary approach, a population of sample points (a sample point is the set of parameters that describe an individual model) is used to generate a new set of points to test, and the subset of points with the best fitness survives to the next generation. In CMA-ES the differences in average fitness between subsequent populations are used to evolve the center of the population toward the optimum. Moreover, knowledge about the interaction between parameters is iteratively gathered in a covariance matrix, which is used to allocate new sampling points so that points are close together in the directions which are well described and further apart in other directions. Because a derivative is never calculated, and just the ranking between solutions is used, this method is resilient to local fluctuations in the fitness landscape.

To simplify model creation, parameter tuning and reproducibility, the parameter optimization algorithm should work with models specified by a declarative model specification. Creation of neuronal models is a time consuming and error prone process, and model code all too often is written in a fashion that impedes reproducibility and extensibility (Gewaltig and Cannon, 2014). A declarative model specification, which separates the model parameters from the simulation itself, e.g., NeuroML (Gleeson et al., 2010; Cannon et al., 2014) or NineML (Raikov et al., 2011; Richmond et al., 2014) simplifies model development and enhances reproducibility. Furthermore, to enhance utility of a parameter optimization algorithm, setting up the optimization and specification of parameters to vary should be independent of the model specification itself.

We describe a versatile software tool, written in Python for the MOOSE simulator (Ray and Bhalla, 2008), for model creation and automatic parameter optimization that can be used by experimentalists and theoreticians alike to automatically fit a model to experimental traces for different neuron types without delving into simulator-specific details.

## Methods

### Overview

We created multi-compartment, multi-conductance models of two neuron types. Table 1 lists the two subtypes of neurons of the external globus pallidus (GPe): arkypallidal neuron (ArkyN) and prototypical neuron (ProtoN); and the two subtypes of striatal neurons: dopamine D1 receptor containing spiny projection neurons (D1-SPN) and dopamine D2 receptor containing spiny projection neurons (D2-SPN). To facilitate model development and inspection, we use a declarative parameter specification to create the models. Python scripts interpret the parameters to create and simulate the multi-compartmental, multi-ion channel model using the MOOSE simulator. For the parameter optimization, the simulated voltage response to current injection is compared to experimentally measured membrane potential using a feature-based fitness function. The parameters are optimized using the covariance matrix adaptation evolutionary strategy (https://github.com/CMA-ES/pycma).

**Table 1:** Types and subtypes of neurons used in the simulations.

**Type**	**Subtypes**	**Names**
Striatal Spiny Projecton (SP) neurons	Dopamine D1 receptor containing spiny projection neuron	D1-SPN
	Dopamine D2 receptor containing spiny projection neuron	D2-SPN
Globus Pallidus (GPe) neurons	Arkypallidal neuron	ArkyN
	Prototypical neuron	ProtoN

### Model specification

To facilitate reproducibility, re-use and extension, the declarative model specification uses a modular format. The ion channel kinetics are specified in one file: (https://github.com/neurord/moose_nerp/blob/master/moose_nerp/d1d2/param_chan.py), e.g.,


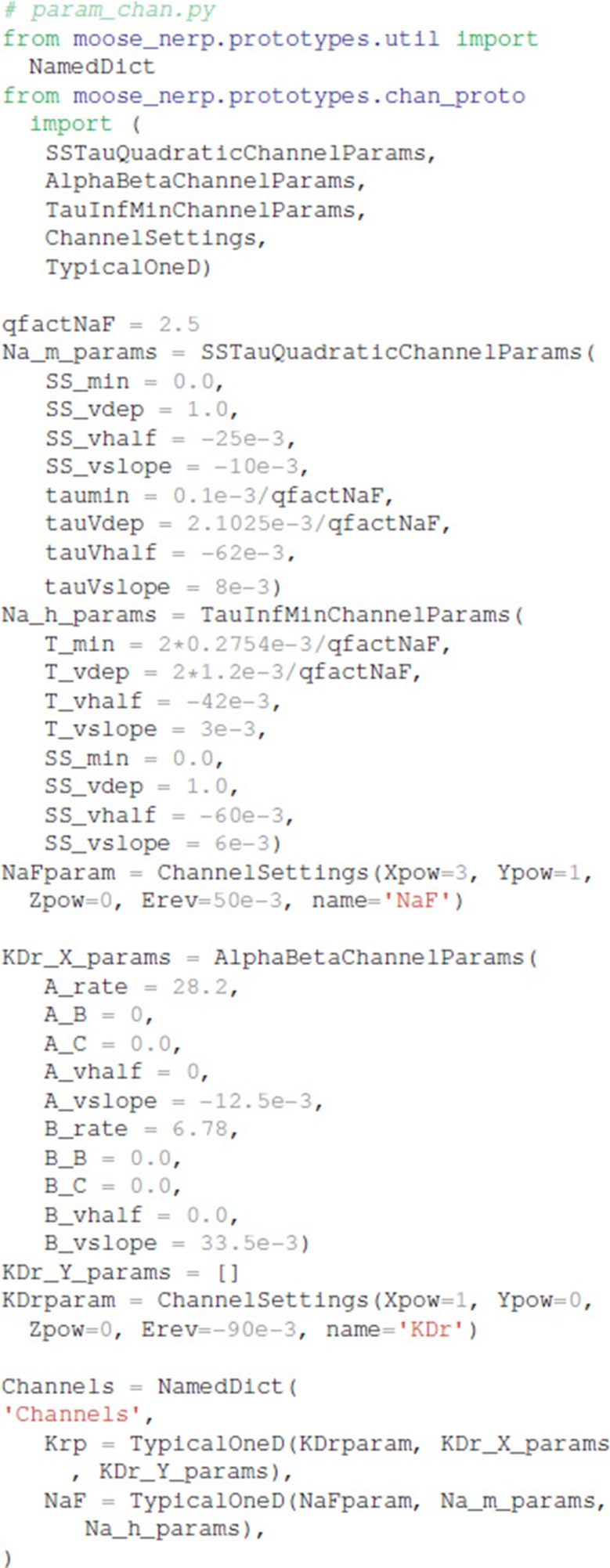


Both the morphology file (either standard GENESIS .p files or .swc files are supported by MOOSE) and conductances (in units of Siemens/m^2^) are specified in a separate file (https://github.com/neurord/moose_nerp/blob/master/moose_nerp/d1d2/param_cond.py), e.g.,


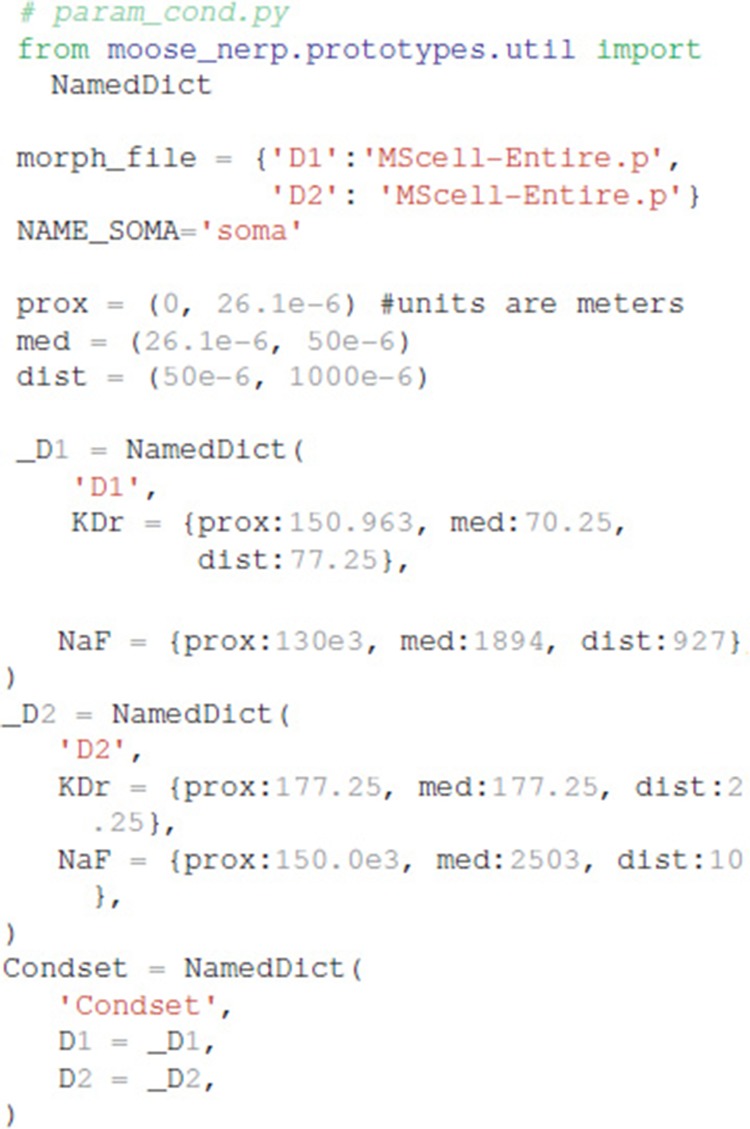


Explicit spines, calcium dynamics, and synaptic channels are each optional and specified in separate files. Calcium dynamics can be specified either with a single time constant of decay or utilizing various mechanisms such as calcium buffers, pumps and diffusion. Model stimulation, creation of output elements and model simulation are clearly and explicitly separated from the model creation. Parameter specification files are imported in https://github.com/neurord/moose_nerp/blob/master/moose_nerp/d1d2/__init__.py:


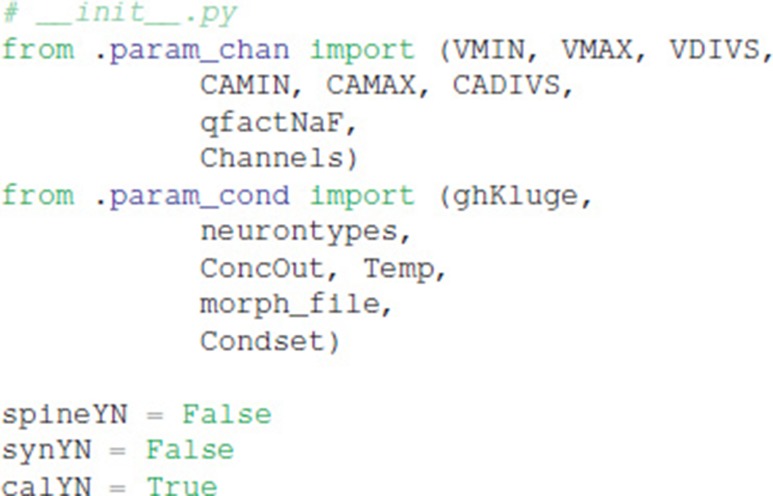


Given these parameter files, the model creation and simulation procedures are implemented in __main__.py, e.g.:


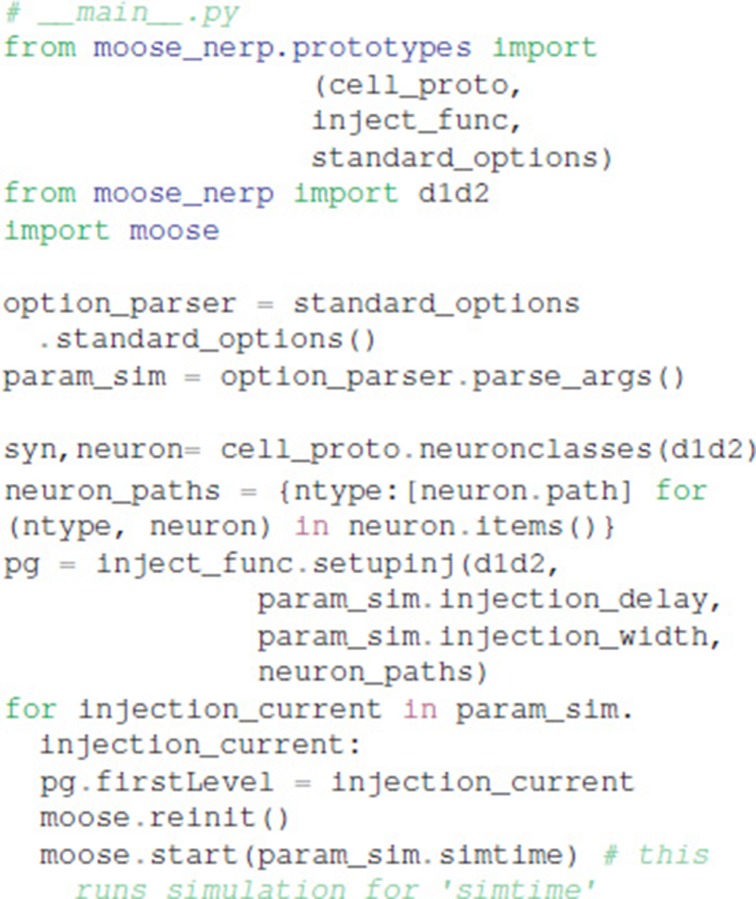


We made the simplifying assumption that both subtypes of GPe neurons had similar kinetics and differed only in channel conductance, as previously suggested (Gunay et al., 2008). Similarly, both subtypes of SP neurons differed only in channel conductance. In contrast, channel kinetics of the GPe neurons differed from that of SP neurons. Models were simulated with PyMoose version 3.1.0 using the hsolve numerical solver. The complete model specification is available at https://github.com/neurord/moose_nerp/, with moose_nerp/d1d2 specifying the SP model parameters and moose_nerp/gp specifying the GPe model parameters.

### Experimental data

All animal handling and procedures were in accordance with the National Institutes of Health animal welfare guidelines and were approved by the George Mason University IACUC committee, or the National Institute on Alcohol Abuse and Alcoholism Animal Care and Use Committee. The experimental data used for the optimizations are part of the python package *waves*, available at https://github.com/neurord/waves. The data consists of recordings from identified external globus pallidus neurons and unidentified striatal spiny projection neurons. As the data was collected for other purposes, the current injection protocol was implemented only once per neuron.

Globus pallidus neuron data was obtained from recordings performed for a prior publication (Abrahao et al., 2017). Briefly, mouse coronal GPe slices, ages P23-P45, were prepared in sucrose cutting solution (in mM: 194 sucrose, 30 NaCl, 4.5 KCl, 26 NaHCO_3_, 1.2 NaH_2_PO_4_, 10 D-glucose, 1 MgCl_2_, and saturated with 95% O2/5% CO2). Slices were equilibrated for 30–40 min at 32°C in carbogen-bubbled aCSF (in mM: 124 NaCl, 4.5 KCl, 26 NaHCO_3_, 1.2 NaH_2_PO_4_, 10 D-glucose, 1 MgCl_2_, and 2 CaCl_2_). Slices were then incubated at room temperature. Recordings were performed at 30–32°C using micropipettes (2–4 MΩ) filled with internal solution (in mM: 140 K-gluconate, 10 HEPES, 0.1 CaCl_2_, 2 MgCl_2_, 1 EGTA, 2 ATP-Mg, and 0.2 GTP-Na, pH 7.25, 300–305 mOsm. Neurons were visualized using an upright microscope (Scientifica, Uckfield, East Sussex, UK) with a LUMPlanFL N × 40/0.80 W objective (Olympus, Waltham, MA). Recordings were obtained using a Multiclamp 700 A amplifier, Digidata 1322 A digitizer and analyzed using pClamp 10.3 software (Molecular Devices, Sunnyvale, CA). A low-pass filter of 2 kHz and sampling frequency of 10 kHz were used. We used the spontaneous firing with no current injection during the 5th min of recording after breakthrough and the response to 1 s hyperpolarizing current injection (from −200 to −50 pA in 50 pA increments). Depolarizing current injection was not used since these neurons fire spontaneously. When recording in slices from wild-type C57BL/6J mice, 1% Neurobiotin (Vector Laboratories, Burlingame, CA) was added into the internal solution for *post hoc* immunocytochemistry of Parvalbumin (PV), a marker for fast spiking prototypical GPe neurons (ProtoN). Though the fast firing, PV+ neurons generally are considered prototypical neurons (four were used for the optimization), the low firing, PV− neurons (three were used for the optimization) are likely a mixture of arkypallidal and other neuron types. Nonetheless, for the purpose of evaluating subtype differences, we are labeling the three low firing, PV− neurons as ArkyN.

Spiny projection neuron data was collected in current clamp from dorso-lateral striatum of *ex vivo* brain slices of C57Bl6 male and female mice, ages P20–P28. Briefly, brain slices were extracted following decapitation of mice anesthetized with isoflurane. Brains were sliced using a VT1000S vibratome (Leica) in oxygenated ice-cold slicing solution (in mM: KCl 2.8, Dextrose 10, NaHCO_3_ 26.2, NaH_2_PO_4_ 1.25, CaCl_2_ 0.5, Mg_2_SO_4_ 7, Sucrose 210). Slices were incubated in aCSF (in mM: NaCl 126, NaH_2_PO_4_ 1.25, KCl 2.8, CaCl_2_, Mg_2_SO_4_ 1, NaHCO_3_ 26.2, Dextrose 11) for 30 min at 33°C, then removed to room temperature (21–24°C) for at least 90 more minutes before use. For whole cell recording, a single hemislice was transferred to a submersion recording chamber (ALA Science) gravity-perfused (at 1–2 ml/min) with oxygenated aCSF containing 50 μM picrotoxin (Tocris Bioscience). Temperature was maintained at 30–32°C (ALA Science) and was monitored with an external thermister. Pipettes were pulled from borosilicate glass on a laser pipette puller (Sutter P-2000) and fire-polished (Narishige MF-830) to a resistance of 3–7 MΩ. Pipettes were filled with a potassium based internal solution (in mM: K-gluconate 132, KCl 10, NaCl 8, HEPES 10, Mg-ATP 3.56, Na-GTP 0.38, Biocytin 0.77) for all recordings. Intracellular signals were collected in current clamp and filtered at 3 kHz using an Axon2B amplifier (Axon instruments), and sampled at 10–20 kHz using an ITC-16 (Instrutech) and Pulse v8.80 (HEKA Electronik). Series resistance (6–30 MΩ) was manually compensated. Voltage responses were collected using 400 ms hyperpolarizing current injection from −500 to −0 in 50 pA increments, and using 400 ms depolarizing current injections, starting from 100 or 200 pA increasing in 20 pA increments. Striatal neurons were identified as being SP neurons (as opposed to fast spiking or low-threshold-spiking interneurons) by their inward rectifier, shallow afterhyperpolarization (AHP), and long latency to fire an action potential in response to current injection. When recording from SP neurons identified using D1Cre- or D2Cre-GFP (green fluorescent protein), the D2Cre-GFP neurons have a lower rheobase current (Chan et al., 2012). Thus, for the purpose of evaluating subtype differences, SP neurons with a rheobase below 200 pA were considered D2-SPN (3 neurons used), and SP neurons with a rheobase above 300 pA were considered D1-SPN (3 neurons used).

### Fitness function

We compared multiple characteristics of spiking and non-spiking activity between simulation and experiment. The spiking characteristics include action potential (AP) time, width, height, number, AHP depth, AHP shape, and (for SP neurons) latency to spike in response to depolarizing current injection. Spike height is calculated with respect to the spike threshold, defined as the point where the membrane potential derivative exceeds 5% of the maximum. Spike height is the difference between spike threshold and the peak membrane potential, and spike width is full width at half height. The non-spiking characteristics include resting potential (both pre- and post-current injection), steady-state voltage response to current injection, time course of membrane potential (falling curve time constant), and rectification (sag caused by inward rectifier, which is the difference between steady state response and the minimum membrane potential deflection during negative current injection). Feature extraction functions are specified in https://github.com/neurord/ajustador/blob/master/ajustador/features.py, and they are combined into a fitness function in https://github.com/neurord/ajustador/blob/master/ajustador/fitnesses.py. To minimize simulation time, for each GPe neuron we used 2 hyperpolarizing traces and 1 trace with no current injection (which contained spontaneously generated action potentials); and for each SP neuron we used 1 hyperpolarizing and 3 depolarizing traces (one of which did not produce action potentials). The difference in feature values between model and data was normalized by dividing by the sum of the model and data response. This normalization converted the feature difference to a fractional, unitless difference. In their multi-objective normalization (Druckmann et al., 2007), divided by the standard deviation of the experimental data. Unfortunately this is not possible for us because our experimental data set is not large enough. We calculated a single fitness value from the weighted sum of the normalized feature differences. A user can further normalize the features by standard deviation by setting the weights equal to the multiplicative inverse of standard deviation, calculated either within neuron if multiple traces are collected or across neurons of a single type. For the simulations reported here, the weights on most features were equal to 1, with several exceptions to produce better fits visually (Table 2 gives weight on each feature, and Figure 2 illustrates experimental and simulated voltage traces for visual inspection of various features).

**Table 2:** Weights on feature values to create fitness function.

**Feature**	**D1-SPN and D2-SPN**	**ArkyN and ProtoN**
Baseline pre	1	0
Baseline post	1	1
Rectification	0	2
Falling curve	1	1
Voltage response	1	1
Latency	1	0
Spike time	0	0.5
Spike width	1	1
Spike height	1	0.5
Spike count	1	1
AHP depth	1	1
AHP curve	4	1
Histogram	1	1

*AHP curve was weighted higher for SPN to avoid the optimizer creating models with large, sharp AHPs. For the GPe models, spike height weight was reduced to avoid the optimizer producing an extreme mismatch in other features while trying to reduce spike height to the unusually small values observed experimentally. Spike time was reduced for both GPe and SPN models reflecting the high variability of this value between neurons of the same type. A weight of zero means to not use the feature, e.g., latency is not defined for a spontaneously spiking neuron. Features are further described in the online documentation (https://neurord.github.io/ajustador/features.html and https://neurord.github.io/ajustador/fitnesses.html). “histogram” is a root-mean-square of the difference between cumulative histograms of membrane potential-values in the two recordings*.

### Parameter optimization

In the optimization loop, the ajustador.optimize. Optimizer class is used as a wrapper for the actual fitting algorithm. Maximum conductances and passive electrical properties may be specified as parameters to vary. Each parameter is assigned an initial value and a permitted range of values (e.g., a minimum value of 0 prevents negative parameters). Appending _0, _1, or _2 to the channel name allows different conductances in the different neuron regions, corresponding to the regions specified in param_cond.py, otherwise the conductance in all neuron regions are made the same value. For the simulations reported, the initial values were conductances from a roughly hand-tuned model to start the optimization in an area that exhibits spiking behavior (Supplementary Figure 1, also available at https://github.com/neurord/ajustador/tree/master/FrontNeuroinf/FigSuppl_initialconditions.jpg). Each neuron of the same type started with the same initial value; thus any differences between neuron subtypes cannot be due to different initial conditions. The CMA-ES loop was started with a high initial estimate of variance, so that a diverse set of parameter values would be explored.


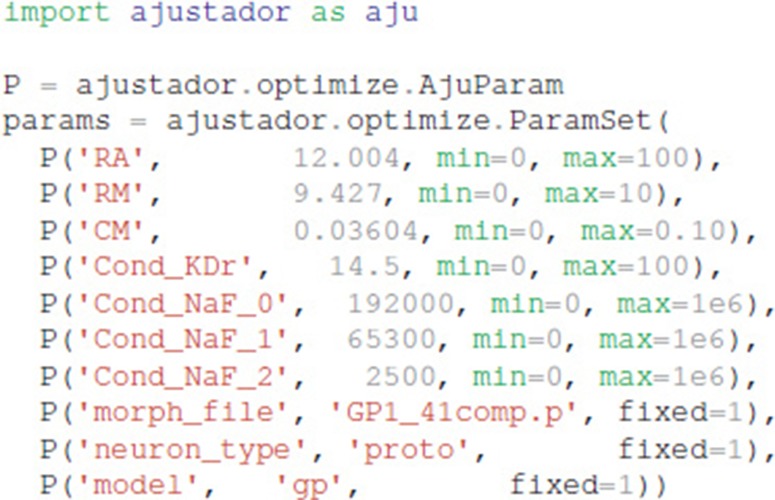


The optimization object uses the specified parameter set, experimental traces, fitness function, and directory for storing the simulation results:


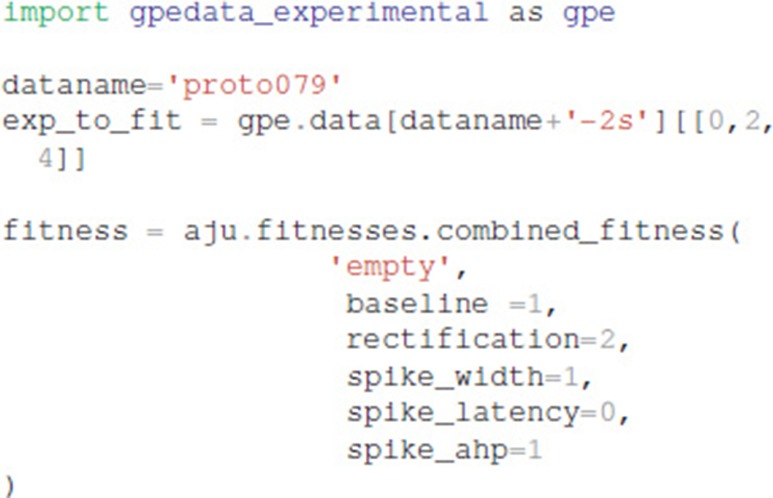


Experimental data can be specified using one of two different file formats: Igor binaries or comma separated values. The traces for the experiments are placed in a separate subdirectory, e.g., gpedata_experimental, and the class Param in the python package waves (https://github.com/neurord/waves) specifies the onset and offset time of the injection current, as well as the time frame for measuring baseline membrane potential and steady state depolarization. Since the data specification is a separate module, adding support for other file formats is straightforward.

It is also necessary to specify which type of model (GPe or SP neurons), which neuron subtype to optimize (e.g., for GPe either arkyN or protoN), and that the simulation is a MOOSE simulation:


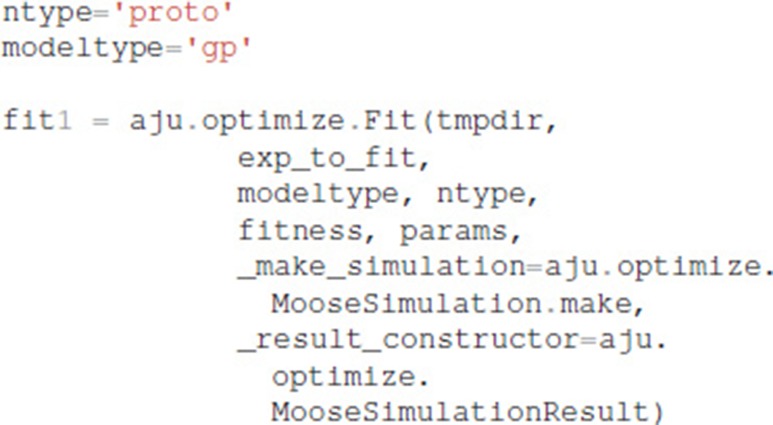


Functions in ajustador.basic_simulation are used by the parameter optimization to run the MOOSE simulation. They implement only the key model creation and simulation commands from __main__.py, thereby simplifying the interface between creation of neuron model and parameter optimization.

After the optimization is configured with this information, the optimization is performed for a specified number of generations, using a specified population size for each generation. The total number of model evaluations is the product of population size and generations. The simulations reported herein used the default population size of 8, but the user can specify other population sizes. Similarly, the user can specify the simulation seed to be used by the do_fit function:


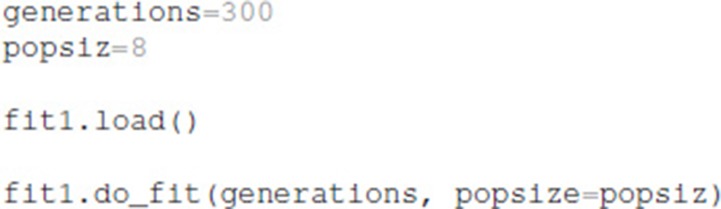


The python package ajustador is available at https://github.com/neurord/ajustador, and the scripts used to run the simulations are at https://github.com/neurord/ajustador/tree/master/FrontNeuroinf.

In many mathematical optimization scenarios, the calculation of the fitness of a single point (individual model) is quick, and the optimization loop is the important part. Here, as often in computational neuroscience, the simulation of each point is a lengthy process, requiring instantiation of the MOOSE interpreter, loading of the model, actual simulation, saving of the result to a file, and finally generating a fitness value from those results. The Optimizer class communicates with the implementation of CMA-ES to retrieve a set of points, perform simulations and calculate the fitness for all of them, feed the results back, so that the numerical algorithm can generate a new set of points to execute. Actual simulation is parallelized at a high level to speed up the whole process: although each individual simulation is single-threaded, during optimization multiple parameter combinations are evaluated together, and for each of those, multiple traces corresponding to different experimental conditions, e.g., different injection currents, need to be simulated. This means that we can take full advantage of available computational power by parallelizing at the level of whole simulations, one simulation per available processor core using a simple queue of jobs. We used Python's multiprocessing module (https://docs.python.org/3/library/multiprocessing.html) to schedule jobs on a single machine, and IPython's ipyparallel (https://ipyparallel.readthedocs.io/en/latest) on multiple machines in a local network. In both cases, the results were saved to disk to a directory with a file containing a copy of the simulation parameters, and files for the simulation results (typically, voltage traces over time). In the multi-machine case a network file system was used to access the storage area. Saving directly to disk provided a mechanism to introspect the running simulation and to retrieve the results for any previously-simulated parameter combination.

When the optimization is complete, the results include the set of parameters, the normalized feature differences, and the overall fitness value for each individual model. The fitness history is the plot of overall fitness value vs. model evaluations (each generation evaluates a population size of models).

To analyze whether parameters are predictive of different neuron subtypes, we used a two-step statistical analysis applied to the parameter values using SAS version 9.4. In step one, a stepwise discriminant analysis was performed (procedure STEPDISC), using the parameter values normalized by standard deviation (procedure STDIZE), to identify the parameters that could perform the best *linear* separation of the two neuron subtypes. In addition, we plotted one parameter value vs. a second parameter value, for all parameters, and inspected these graphs to visualize which parameters segregated and clustered the two neuron subtypes. In step two, a cluster analysis was performed using those variables identified in step one, to assess the extent to which the neuron subtypes segregated. Two methods of cluster analysis were performed. First the procedure CLUSTER was used to determine the optimum number of clusters. Then, the procedure FASTCLUS was used, on the data normalized with STDIZE and with the number of clusters determined by CLUSTER, to calculate the distance between clusters of same and different neuron subtypes. The procedure FREQ was appied to the output of the cluster analysis to generate the confusion matrices.

## Results

### Declarative model specification

We created a python module called moose_nerp (moose neuron prototype) to simplify and standardize the creation and simulation of neuron models using the MOOSE software. The declarative framework facilitates reproducibility, re-use and extension of MOOSE models of neurons and networks. Each set of neuron models has a set of parameter files specifying (1) channel kinetics, (2) channel conductances and morphology, (3) synaptic channel parameters, (4) calcium mechanism parameters, and (5) spine parameters. Spines, synapses and calcium dynamics can be included or excluded with a simple parameter switch, e.g., calYN = True and spineYN = False. Parameter specifications for channel kinetics and conductances use similar organization, keywords and parameter types as NeuroML version2, facilitating conversion, whereas the parameters for calcium dynamics, such as buffer and pump specifications, do not yet have NeuroML version2 equivalents.

Two subtypes of each of two types of neuron models were created for use with the parameter optimization. Models of the two subtypes of neurons in the globus pallidus were developed, representing arkypallidal (low firing rate, PV−, ethanol sensitive) and prototypical (high firing rate, PV+, ethanol insensitive) by creating a set of parameter files. In addition, models of the two subtypes of striatal spiny projection neurons in the striatum were developed (called D1-SPN and D2-SPN, representing the direct pathway neurons that contain dopamine D1 receptors and the indirect pathway neurons that contain dopamine D2 receptors) by creating a second set of parameter files specifying channel kinetics, conductances, etc. Channel kinetics for the GPe neuron models were adapted from Hendrickson et al. (2011a); both arkyN and protoN neurons used the same channel kinetics and morphology. Channel kinetics for the SP neuron models were adapted from Jedrzejewska-Szmek et al. (2017); both D1-SPN and D2-SPN used the same morphology and channel kinetics. Both models used single time constant of decay for calcium dynamics, though calcium buffers, pumps and diffusion have been implemented in the SP neuron models and can be specified with a parameter switch.

### Parameter optimization using CMA-ES

**Figure 1:**
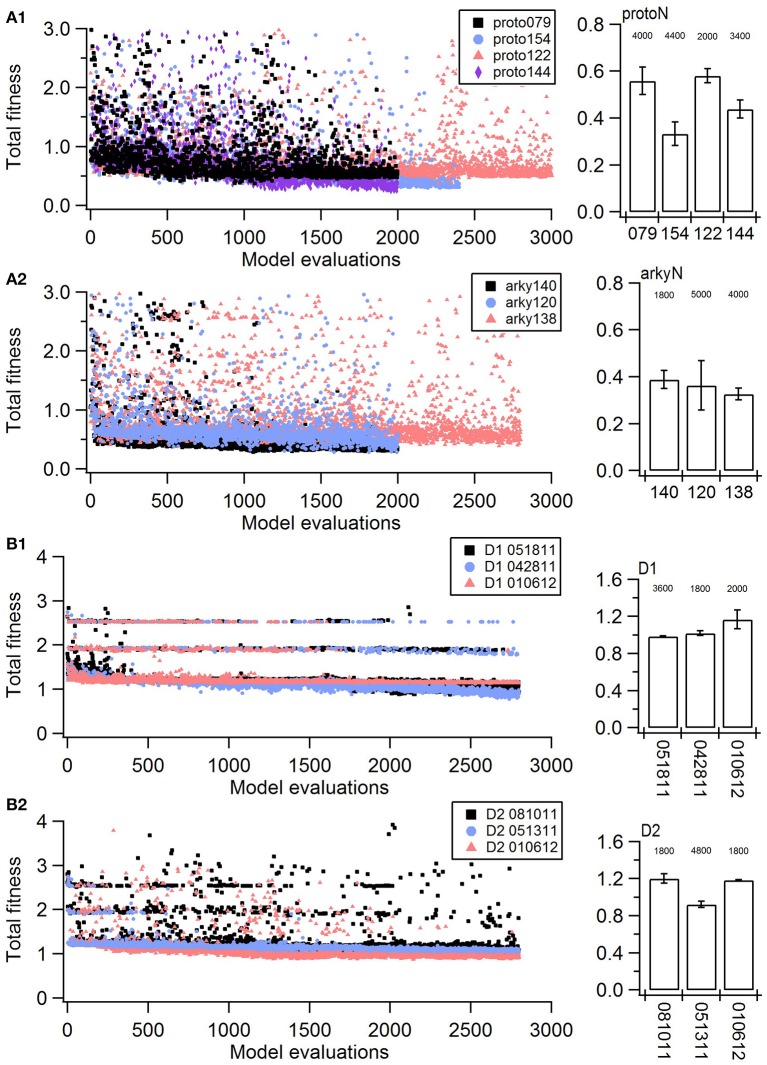
Fitness history shows the fitness values rapidly reach good fits (within 1,000 model evaluations/sample points) and reaches an asymptote typically within 2,000 model evaluations/sample points). **(A)** Fitness vs. model evaluation for GPe neurons of **(A1)** prototypical type and **(A2)** arkypallidal type. **(B)** Fitness vs. model evaluation for SP neurons of **(B1)** D1 type and **(B2)** D2 type. Note that GPe neuron fitness values reached considerably lower values than SP neuron fitness values. Right panels show fitness vs. model evaluation for the 1st set of optimizations and left panels show the mean and standard deviation of the fitness values of the last 25 generations of the 2nd set of optimizations (which used a different random seed). The number above the bar gives the number of model evaluations to convergence for the 2nd set of optimizations.

Parameter optimization was run on a 16-core Linux workstation with Intel^Ⓡ^ Xeon^Ⓡ^ CPU E5-2650 processors. Each of the four neuron models was optimized to 3–4 sets of voltage traces, each set from a different, experimentally recorded neuron. For each recorded neuron, traces both with and without action potentials were utilized in a single optimization. Optimizations were run until the fitness value reached an asymptote, typically within 200–500 generations using a population size of 8 (Figure 1, Table 3). The convergence was determined from the change in mean fitness: the slope of the mean fitness across 25 generations must be < 0.002 and the standard deviation of mean fitness across 25 generations must be less than 0.06 (implemented in https://github.com/neurord/ajustador/tree/master/ajustador/helpers/converge.py). For each optimization, all current injections are simulated in parallel. In general, each simulation job is submitted to a scheduler, and started when resources are available. The result is returned to the optimization algorithm when all requested points have been finished.

**Table 3:** Characteristics of optimization simulations.

	**SP**	**GPe**
Number of compartments	189	41
Number of experimental traces used	4	3
Duration of trace	0.9 s	2 s
Simulation time^*^	12.4 ± 1.03 h per 1,000 models	8.5 ± 0.16 h per 1,000 models
Evaluations to convergence^#^	2,100 ± 500	3,514 ± 1,007
Evaluations till fitness value is within 5% of minimum	867 ± 1,185	2,482 ± 1,133

* *Reported simulation time (mean and standard deviation) is for the second simulation seed*.

# *Evaluations to convergence (mean and standard deviation) is for the second simulation seed; one of the GP simulations did not reach convergence within 5,000 model evaluations, yet reached a minimum fitness of 0.26*.

The parameter algorithm was able to find reasonable parameters for most of the data sets. We defined the feature funtions in a way that would give values on the order of one, so that when multiple features were combined with equal weights, all features could contribute significantly to the total. The optimizations were originally performed using equal weighting, and then repeated once or twice after visual comparison of simulations and experiments and adjusting the weights (Table 2) to de-emphasize spike time and improve the fit to shape of the AHP. Figure 1 shows total fitness value vs. model evaluation for GPe neurons (A) and SP neurons (B). Most combined fitness values decreased to ~0.4 or less for the seven GPe neurons and to ~1.0 or less for the SP neurons. Simulations were repeated using a different random seed, with similar results: the change in minimum total fitness reached was 0.018 (6.4%) for GPe neurons and −0.041 (4.4%) for SP neurons.

**Figure 2:**
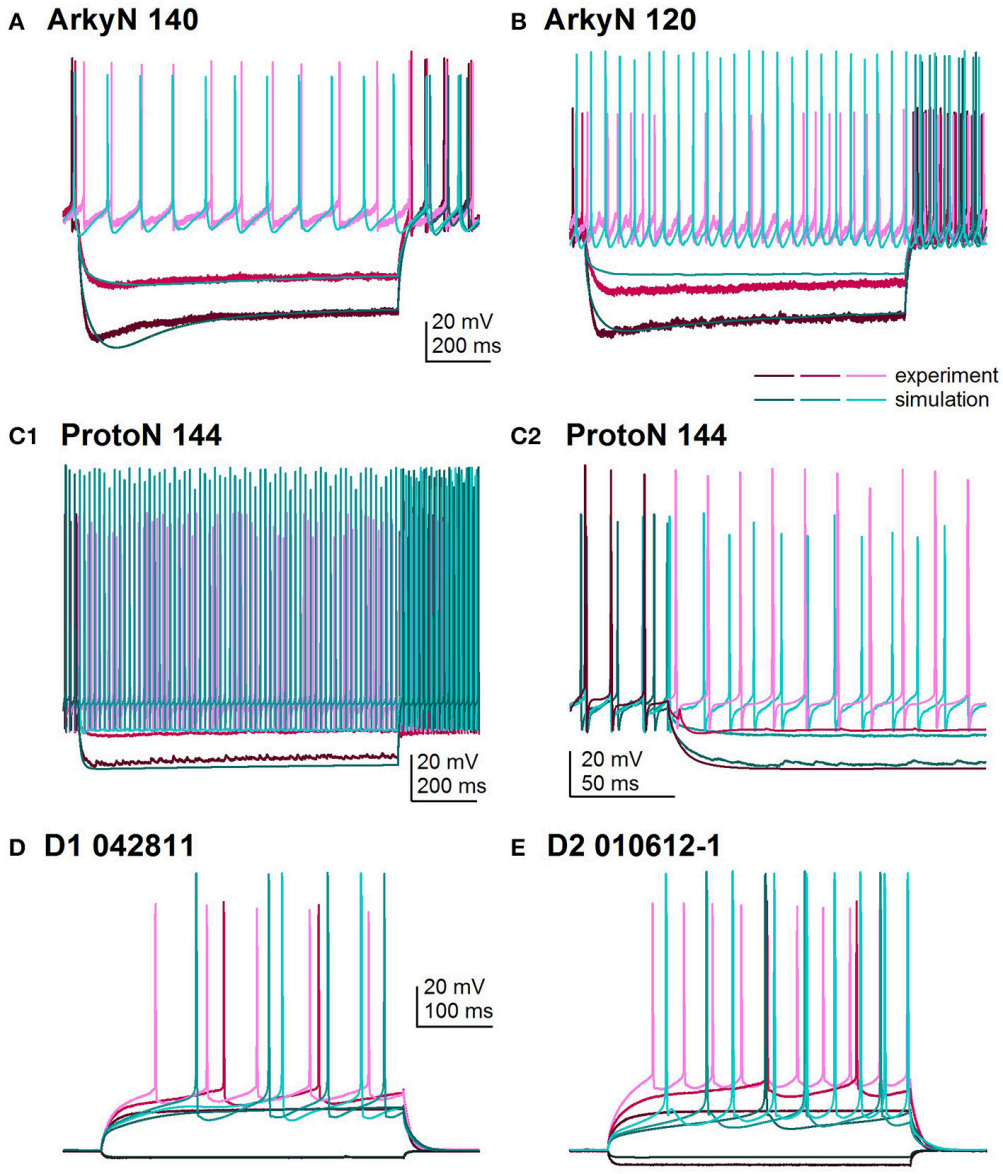
Comparison of simulated and experimental traces. In all panels, simulations are in shades of turquoise and experimental data in shades of magenta. **(A)** Fit to arkypalidal cell #140 (minimum fitness = 0.29). Spike height, timing and AHP are all fit quite well. **(B)** Fit to arkypallidal cell #120 (minimum fitness = 0.29). This example shows the difficulty in fitting to spike height when spikes are shorter than usual. **(C)** Fit to protoypical cell #144 (minimum fitness = 0.25). C1 shows fit to entire 1 sec of current injection, whereas C2 zooms in to illustrate match to AHP shape. **(D)** Fit to D1R type of SP neuron (minimum fitness 0.78). **(E)** Fit to D2R type of SP neuron (minimum fitness 0.88). Both **(D,E)** show good fit to AP shape, AHP shape and long latency to fire.

Figure 2 shows an overlay of the model traces and experimental data for the optimizations in Figure 1 to illustrate similarity between model and experiments. Figures 2A,B show optimizations of two different arkypallidal neurons from the external globus pallidus. For both neurons, the shape of the AHP and the amplitude of the sag match quite well. On the other hand, the fit to arky N 120 shows the difficulty in fitting to neurons with short action potentials (similar results are obtained with a spike height weight of 1.0). Figure 2C shows the fit to a prototypical neuron, which fires at a much faster rate than the arkypallidal neurons. The ability to match the shape of the AHPs is illustrated in Figure 2C2 which expands the time scale of the plot. Figures 2D,E show optimizations of one D1-SPN and one D2-SPN. Again, AP characteristics and AHP shape fit quite well.

**Figure 3:**
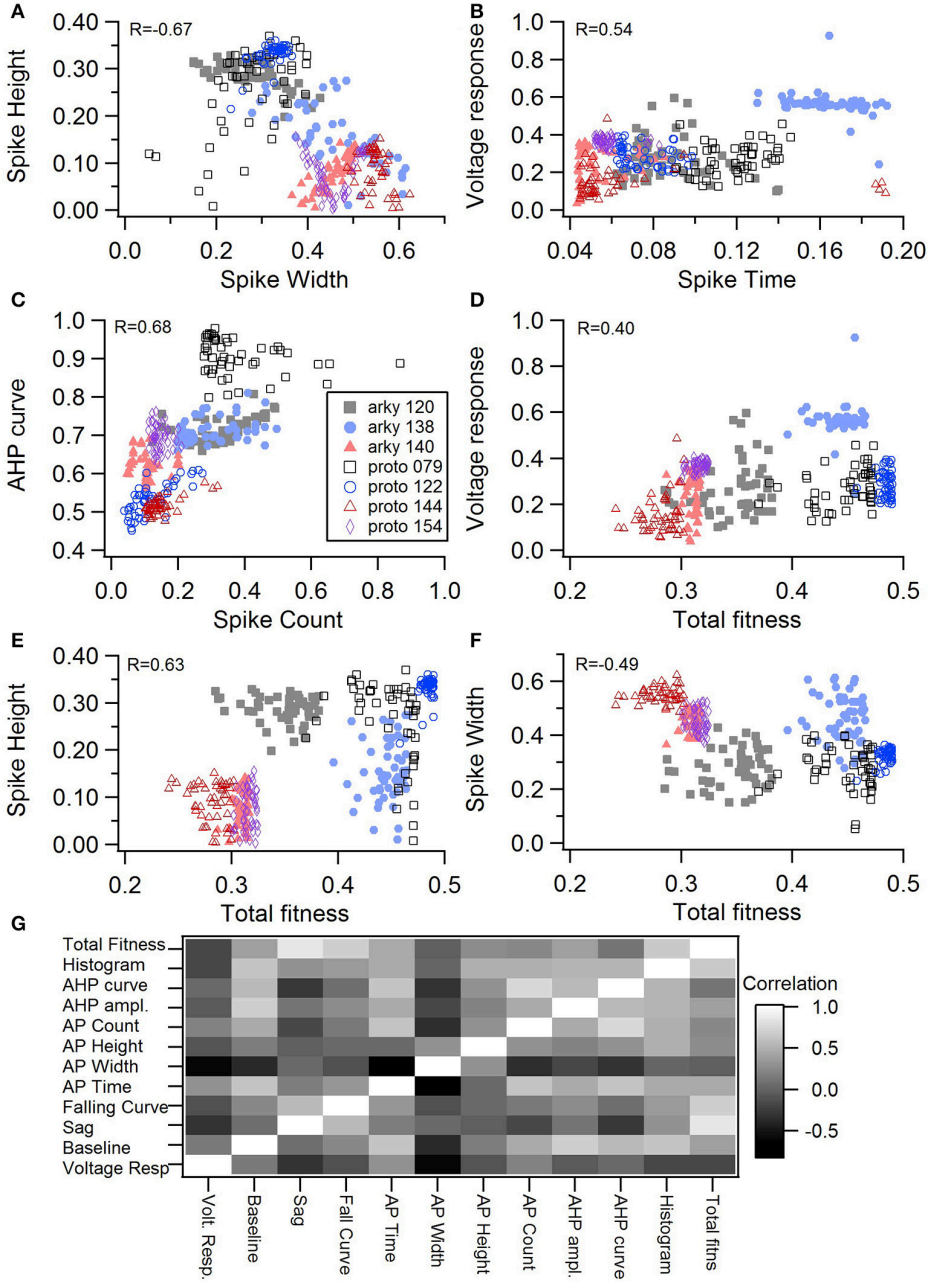
Comparison of feature fitnesses for 50 best models for GPe neuron optimizations. **(A)** Spike height vs. spike width shows that improvements in spike height come at expense of worsening of spike width. In contrast to this trade-off, **(B)** steady state voltage response vs. spike time and **(C)** AHP curve vs. spike count show that two features can improve simultaneously. **(D–E)** contribution of voltage response **(D)**, spike height **(E)** and spike width **(F)** to the total fitness. Despite the significant positive correlation for two of the features, no one feature appears to control the fit. R is the Pearson's R correlation; all illustrated correlations are significant at *P* < 0.0001. Symbols corresponding to different neurons are the same in all panels and indicated in **C**. **(G)** Pairwise Pearson's R correlation between all features illustrated as image plot. AP: spike.

**Figure 4:**
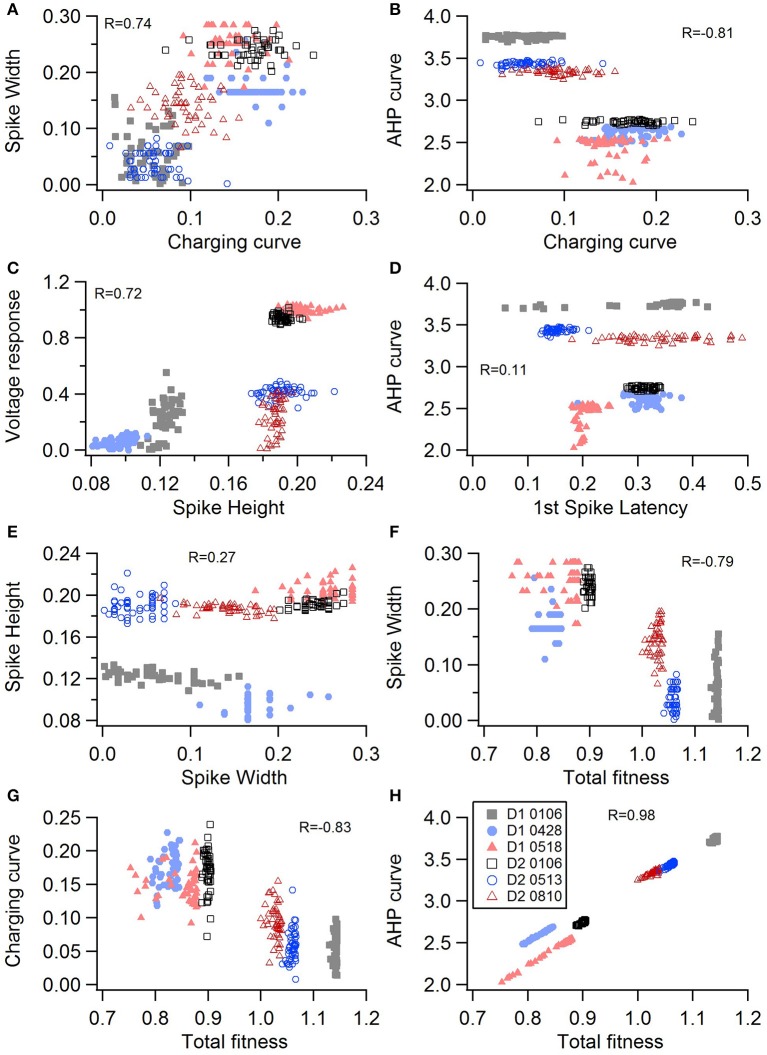
Comparison of feature fitnesses for 50 best models for SP neuron optimizations. The positive correlations for **(A)** spike width vs. charging curve fitness and **(C)** voltage response vs. spike height show that two features can improve simultaneously. With some features, such as **(B)** AHP curve vs. charging curve fitness, there is a trade-off between these two features.**(D)** AHP curve is not correlated with 1st spike latency (*P* = 0.052). **(E)** Spike height vs. spike width does not appear to be correlated in the SP neurons, though reaching statistical significance (*P* < 0.0001). **(F–H)** Contribution of spike width **(F)**, charging curve **(G)**, and AHP curve **(H)** to the total fitness. Improvement in spike width is negatively correlated with total fitness. The strong correlation of AHP curve to total fitness is likely caused by strong weight on AHP curve in the fitness function. R is the Pearson's R correlation; Correlations above 0.7 are significant at *P* < 0.0001. Symbols corresponding to different neurons are the same in all panels and indicated in **H**.

One motivation for using a multi-objective optimization is the observation that improvement in the fit of one feature often comes at the expense of another feature (Druckmann et al., 2007; Rumbell et al., 2016; Neymotin et al., 2017). To evaluate to what extent this trade-off occurs in these single objective optimizations, we evaluated the correlation between various feature functions for the 2.5% best fitting (lowest total fitness value) models (or the last 50 of the best fitting models if more than 2,000 evaluations were performed). The feature fitnesses and total fitness value for (mean over the 50 best models) for each data set is provided in Tables 4A,B. Figures 3A–C, 4A–E shows that very few trade-offs are evident between the features that comprise the fitness function. For the GPe neurons, spike height improves as spike width worsens, but this relationship does not hold for the SP neurons (Figure 4E). Several positive correlations are notable. An increase in AHP curve fitness is correlated with an increase in spike count fitness (Figure 3C), and an increase in voltage response fitness is correlated with an increase in spike time fitness (Figure 3B) for GPe neurons. For the SP neuron optimization, trade-offs are less apparent, and instead the charging curve fitness is positively correlated with the spike width fitness (Figure 4A), though negatively correlated with AHP curve (Figure 4B) fitness. In addition, the voltage response fitness is positively correlated with spike height fitness (Figure 4C). As the long latency to 1st spike in SP neurons is attributed to transient potassium currents, which also can produce large AHPs, we examined AHP curve vs. 1st spike latency (Figure 4D), but the correlation between these two features is quite small. Graphs of single features vs. total fitness (Figures 3D–F, 4F–H) demonstrate that most single features are either not correlated with the total fitness, or explain very little of the variance, e.g., voltage response for the GPe neurons (Figure 3D), and spike latency (*R* = 0.05) and spike count (*R* = −0.31) for SP neurons. A summary of all correlations is provided for GPe neurons in Figure 3G. The lack of correlation reflects that the total fitness is calculated from the combination of multiple features. An exception to this is the high correlation between AHP curve and total fitness for SP neurons, likely due to the high weight of this feature in the total fitness (Figure 4H). Curiously, in some cases feature fitness is negatively correlated with total fitness, such as spike width for both GPe neurons (Figure 3F) and SP neurons (Figure 4F), and charging curve for SP neurons (Figure 4G).This shows that strong fitting of a specific feature can result in a model that is weak when other features are considered, possibly because the model is not flexible enough to provide a good fit on all of those features.

**Table 4A:** Mean feature fitnesses of the 50 best models for each of the globus pallidus neurons.

**GPe**	**proto154F**	**proto144F**	**proto122F**	**proto079F**	**arky140F**	**arky138F**	**arky120F**
Voltage response	0.367 ± 0.022	0.154 ± 0.085	0.289 ± 0.057	0.272 ± 0.081	0.240 ± 0.093	0.565 ± 0.076	0.273 ± 0.116
Baseline post	0.086 ± 0.034	0.093 ± 0.050	0.084 ± 0.047	0.125 ± 0.098	0.050 ± 0.036	0.066 ± 0.049	0.079 ± 0.055
Rectification	0.261 ± 0.107	0.324 ± 0.130	1.318 ± 0.015	0.705 ± 0.267	0.540 ± 0.037	0.853 ± 0.146	0.511 ± 0.241
Falling curve	0.222 ± 0.090	0.132 ± 0.074	0.298 ± 0.075	0.321 ± 0.173	0.227 ± 0.037	0.256 ± 0.113	0.155 ± 0.087
Spike time	0.058 ± 0.007	0.065 ± 0.038	0.075 ± 0.011	0.118 ± 0.015	0.052 ± 0.011	0.164 ± 0.015	0.087 ± 0.019
Spike width	0.450 ± 0.042	0.548 ± 0.028	0.325 ± 0.027	0.277 ± 0.077	0.455 ± 0.036	0.464 ± 0.089	0.278 ± 0.069
Spike height	0.075 ± 0.042	0.089 ± 0.042	0.332 ± 0.025	0.258 ± 0.089	0.077 ± 0.036	0.156 ± 0.065	0.287 ± 0.029
Spike count	0.144 ± 0.023	0.148 ± 0.047	0.121 ± 0.062	0.378 ± 0.121	0.103 ± 0.039	0.306 ± 0.087	0.326 ± 0.088
AHP amplitude	0.070 ± 0.033	0.112 ± 0.052	0.104 ± 0.047	0.115 ± 0.084	0.074 ± 0.035	0.104 ± 0.077	0.094 ± 0.083
AHP curve	0.693 ± 0.032	0.516 ± 0.022	0.534 ± 0.042	0.904 ± 0.046	0.616 ± 0.033	0.714 ± 0.032	0.712 ± 0.033
Histogram	0.299 ± 0.039	0.239 ± 0.092	0.329 ± 0.049	0.478 ± 0.110	0.146 ± 0.052	0.332 ± 0.096	0.271 ± 0.074
Total	0.316 ± 0.007	0.281 ± 0.015	0.484 ± 0.005	0.448 ± 0.026	0.310 ± 0.006	0.445 ± 0.016	0.348 ± 0.026

**Table 4B:** Mean feature fitnesses of the 50 best models for each of the striatal spiny projection neurons.

	**D1_051811**	**D1_042811**	**D1_010612**	**D2_081011**	**D2_051311**	**D2_010612**
Voltage response	0.996 ± 0.021	0.057 ± 0.032	0.228 ± 0.121	0.243 ± 0.121	0.414 ± 0.038	0.944 ± 0.025
Baseline pre	0.018 ± 0.001	0.072 ± 0.003	0.044 ± 0.007	0.110 ± 0.026	0.015 ± 0.001	0.073 ± 0.001
Baseline post	0.016 ± 0.001	0.057 ± 0.002	0.039 ± 0.008	0.004 ± 0.003	0.013 ± 0.001	0.061 ± 0.001
Falling curve	0.233 ± 0.039	0.058 ± 0.026	0.273 ± 0.084	0.393 ± 0.053	0.294 ± 0.111	0.076 ± 0.018
Spike width	0.253 ± 0.028	0.171 ± 0.022	0.055 ± 0.040	0.141 ± 0.030	0.040 ± 0.022	0.241 ± 0.016
Spike height	0.203 ± 0.008	0.096 ± 0.007	0.123 ± 0.005	0.187 ± 0.004	0.191 ± 0.010	0.191 ± 0.004
Spike latency	0.207 ± 0.017	0.311 ± 0.027	0.332 ± 0.086	0.339 ± 0.070	0.153 ± 0.017	0.313 ± 0.016
Spike count	1.077 ± 0.011	1.066 ± 0.001	1.021 ± 0.067	0.958 ± 0.044	0.946 ± 0.000	0.908 ± 0.004
AHP amplitude	0.187 ± 0.015	0.019 ± 0.013	0.342 ± 0.003	0.184 ± 0.014	0.256 ± 0.003	0.170 ± 0.013
AHP curve	2.434 ± 0.147	2.618 ± 0.062	3.750 ± 0.023	3.336 ± 0.032	3.437 ± 0.025	2.744 ± 0.019
Charging curve	0.147 ± 0.024	0.174 ± 0.025	0.056 ± 0.022	0.094 ± 0.026	0.058 ± 0.023	0.170 ± 0.030
Histogram	0.591 ± 0.011	0.075 ± 0.007	0.357 ± 0.019	0.392 ± 0.036	0.601 ± 0.010	0.441 ± 0.009
Total	0.851 ± 0.036	0.826 ± 0.016	1.142 ± 0.003	1.027 ± 0.0093	1.060 ± 0.006	0.899 ± 0.004

**Figure 5:**
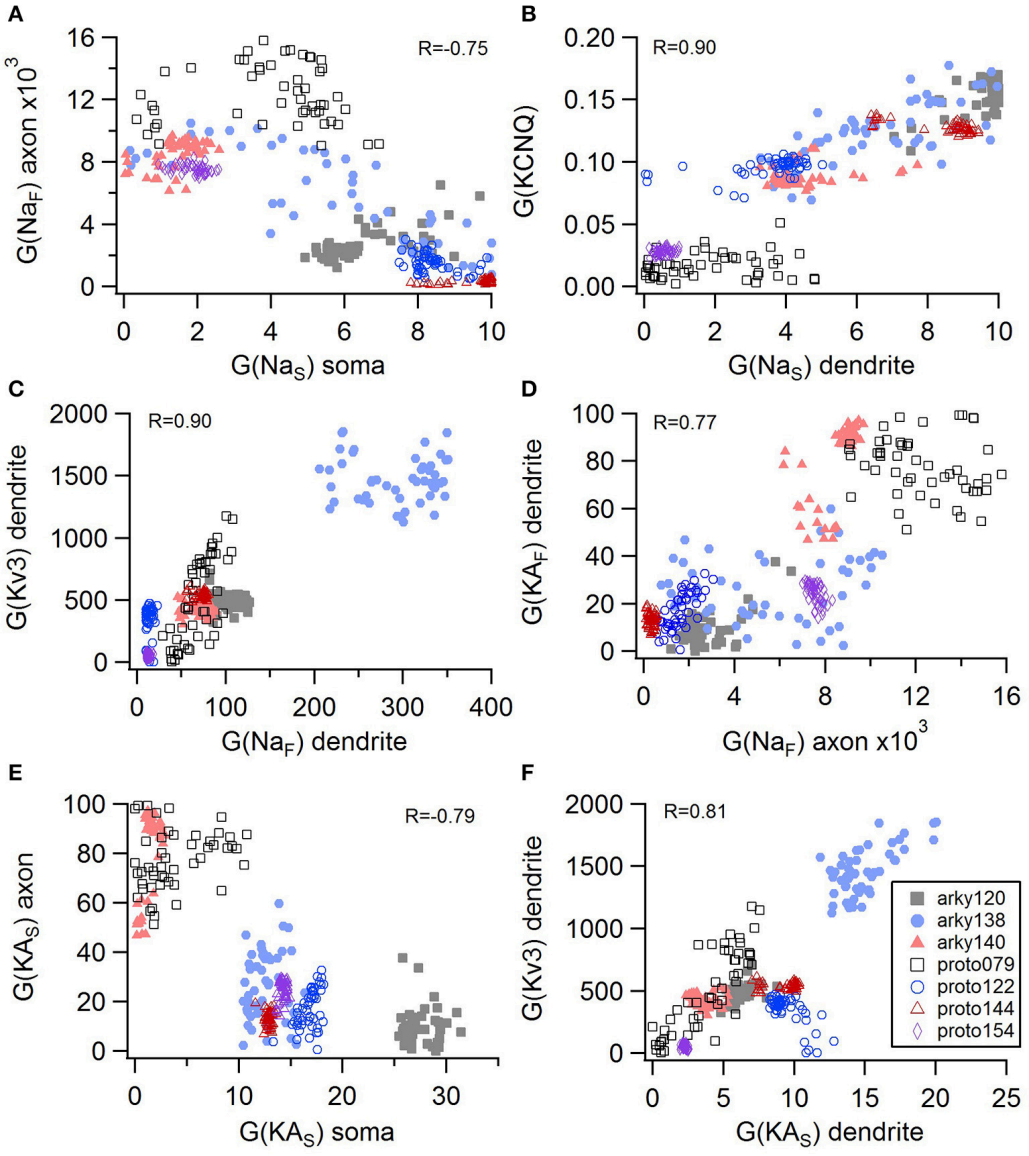
Compensation and other correlations among channel conductances for GPe neurons. **(A)** A decrease in fast sodium conductance in the axon can be compensated by an increase in the slow sodium conductance in the soma. **(B–D)** An increase in conductance of various potassium channels can be compensated by an increase in sodium conductance. **(E)** An increase in the transient potassium current in the soma is correlated with a decrease in the axon. **(F)** A non-compensatory correlation: an increase in KA_S_ type of potassium conductance is associated with an increase in the Kv3 potassium conductance. R is the Pearson's R correlation; all illustrated correlations are significant at *P* < 0.0001. Symbols corresponding to different neurons are the same in all panels and indicated in **F**.

**Figure 6:**
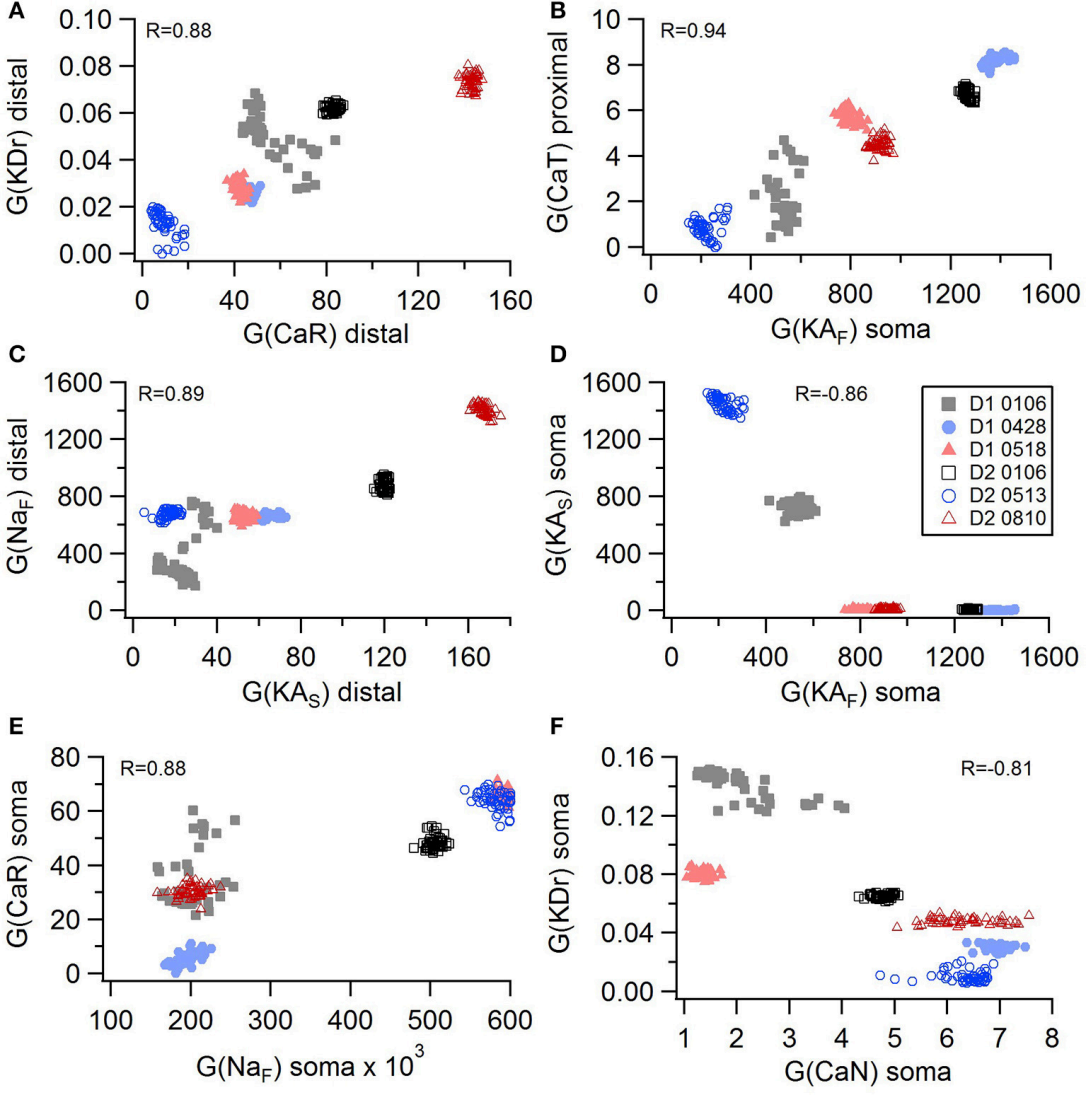
Compensation and other correlations among channel conductances for SP neurons. **(A–C)** An increase in potassium channel conductance is compensated by an increase in inward channel conductance (sodium or calcium channels). **(D)** An increase in fast transient (KA_F_) potassium channel conductance is compensated by a decrease in slow transient (KA_S_) potassium channel conductance in in the soma. **(E,F)** Two non-compensatory correlations between channel conductances. **(E)** An increase in the fast sodium current in the soma is associated with an increase in the R type calcium channel (CaR) conductance. **(F)** An increase in delayed rectifier potasium channel is correlated with a decrease in N type calcium current in the soma. R is the Pearson's R correlation; all illustrated correlations are significant at *P* < 0.0001. Symbols corresponding to different neurons are the same in all panels and indicated in **E**.

Non-linear systems are often difficult to find parameters for because a unique set of parameters may not exist. Prior studies (Golowasch et al., 2002; Prinz et al., 2003a) have observed that higher outward conductances can be compensated by higher inward, or different potassium conductances can compensate for each other. To examine to what extent this occurs in our optimizations, we evaluated the correlation between the different conductances from the same best models as used above. Figure 5 illustrates the conductances for the best GPe models and demonstrates several types of compensation. In the GPe neurons, an increase in the slow sodium current (Na_S_) is compensated by a decrease in the fast sodium current (Na_F_) in the axon (Figure 5A) or an increase in the KCNQ potassium current (Figure 5B). Similarly, an increase in the fast sodium current is compensated by an increase in the Kv3 potassium current (Figure 5C) or an increase in the fast transient potassium (KA_F_) current (Figure 5D). There is a tradeoff between somatic and axonal transient potassium (KA_S_) currents (Figure 5E). In contrast to these compensatory correlations, Figure 5F demonstrates a non-compensatory correlation: the dendritic KA_S_ current positively correlates with the dendritic Kv3 current. A similar range of correlations is apparent for the SP optimizations (Figure 6). Figures 6A–C shows inward currents compensating for outward currents. Figure 6D shows the slow transient potassium current (KA_S_) compensating for the fast transient potassium current (KA_F_) in the soma; whereas Figures 6E,F shows non-compensatory correlations: A correlated increase in two inward currents (Figure 6E), or a decrease in a calcium current correlated with an increase in a potassium current (Figure 6F).

### Approach to identifying mechanisms underlying difference between cell types

CMA outputs provide parameters for generating sets of good models instead of the parameters for the single best fit model. This has the advantage of providing sets of good models for performing simulation experiments and demonstrating robustness to parameter variations. In addition, the parameters themselves can be analyzed to determine whether certain parameters are predictive of different cell types and capture the feature differences between neuron subtypes (Table 5). To address this latter question, we used a multi-step statistical analysis (discriminant analysis followed by cluster analysis) applied to the 50 best fitting models.

**Table 5:** Mean feature properties of data.

	**arky (*N* = 3)**	**proto (*N* = 4)**
AP count	33.67 ± 14.50	134.25 ± 38.66
Spike Height	0.0623 ± 0.0094	0.0675 ± 0.0100
Spike Width	0.00045 ± 0.00008	0.00028 ± 0.00008
Spike AHP	−0.0506 ± 0.0028	−0.0581 ± 0.0040
Baseline Vm	−0.0446 ± 0.0023	−0.0496 ± 0.0044
Rectification (at −200 pA)	0.00787 ± 0.00289	0.00298 ± 0.00299
deltaV (at −100 pA)	−0.0272 ± 0.0039	−0.0114 ± 0.0043
deltaV (at −200 pA)	−0.0430 ± 0.0068	−0.0229 ± 0.0029
Falling curve	0.0129 ± 0.0022	0.0072 ± 0.0014

*Distinguishing features include number of action potentials, spike width, deltaV (input resistance), falling curve*.

**Figure 7:**
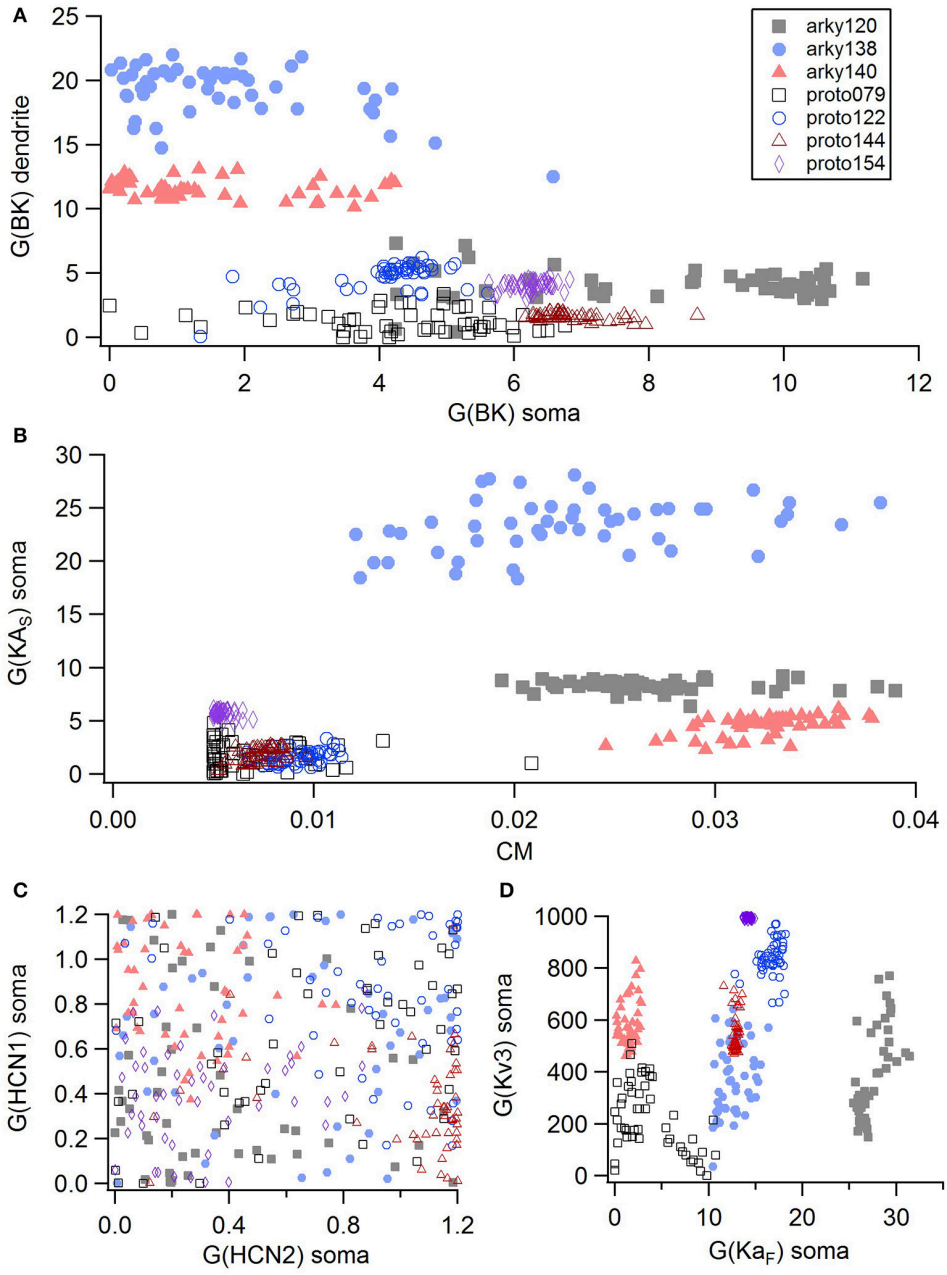
A small number of parameters separate the two subtypes of GPe neurons. **(A)** The large conductance, calcium dependent potassium conductance (BK) in soma and dendrite are larger in arkyN than in protoN. **(B)** Capacitance (CM), and (to a lesser extent) the slow transient potassium channel conductance are greater in arkyN than in protoN **(C–D)** No systematic differences are observed in HCN conductance **(C)** or in Kv3 or KA_F_ **(D)** between arkyN and protoN. Symbols corresponding to different neurons are the same in all panels and indicated in **A**.

For the GPe neurons, graphical analysis revealed that capacitance (CM) and the large conductance, calcium dependent potassium current (BK) in soma and dendrite as the variables that best separate the data. The discriminant analysis similarly identified capacitance, but did not identify the BK conductance. Instead, it identified the slow transient potassium current (KA_S_) in the soma. A plot of these parameter values (Figures 7A,B) demonstrate that the arkyN have either a higher somatic or dendritic BK conductance, and also have a higher capacitance. Inspection of the panels in Figures 5, 7C,D confirm that most of the other parameters do not separate the data by neuron class.

We performed a cluster analysis using these identified parameters (CM and either BK or KA_S_). Because the BK conductance was elevated in either the soma or the dendrite, but not always both, we used the sum of the somatic and dendritic BK conductance as one of the variables. Two types of cluster analyses were performed. The first analysis used the SAS CLUSTER procedure, which performs a hierarchical cluster analysis without the need to specify either the number of clusters or the cluster size. This procedure provides a measure of the goodness of separation vs. number of clusters. Using the number of clusters suggested by the 1st cluster analysis, the second cluster analysis, which implements a disjoint cluster analysis using the SAS FASTCLUS procedure, then provides a measure of the distance between the clusters. This second procedure allowed quantification of the difference between neuron subtypes.

The disjoint cluster analysis using the 3 clusters suggested by the hierarchical cluster analysis correctly classifies all but two of the neuron parameter sets correctly (Table 6), regardless of whether BK or KA_S_ was used. This suggests that the parameters identified may represent subtype differences. The BK conductance in particular has already been demonstrated to differ between arkypallidal and prototypical GPe neurons. Because the parameter optimizations used the same morphology for arkyN and protoN, the difference in CM values suggests that the morphology of these two neurons differ, with arkypallidal neurons having either a larger number of dendrites or a greater number of spines. The greater conductance of the slow transient potassium channel may be producing the shallower AHPs in arkyN as compared to protoN (e.g., compare Figure 2B with Figure 2C2). The Euclidean distance between centroids of the two arkyN clusters (1.78) is smaller than the distance between centroids of the arkyN and protoN clusters (2.95 and 2.27). When the analysis was repeated on the best models from the second set of GPe optimizations, a similar KA_S_ conductance was identified, but instead of the CM or the BK conductance, KA_F_, Na_F_, and KDr were identified. The difference in these two sets of variables suggests that a larger set of optimizations is needed (with fewer models per optimization) for accurate identification of differing channel conductances.

**Table 6:** Confusion matrix for cluster analysis using CM and total BK conductance.

**Cell**	**Cluster 1 (protoN)**	**Cluster 2 (arkyN)**	**Cluster 3 (arkyN)**
Arky120	1^*^	48	1
Arky138	0	0	50
Arky140	0	50	0
Proto079	50	0	0
Proto122	50	0	0
Proto144	50	0	0
Proto154	50	0	0

*Note that labeling the clusters was performed post-hoc, based on the composition of the clusters*.

* *Indicates the incorrectly classified parameter set*.

## Discussion

We created python code for automatic parameter optimization of single neuron models simulated using the MOOSE software. In order to facilitate development and reuse of multi-compartment, multi-conductance models, we used a declarative parameter specification to create the models, and then demonstrated its utility by creating two subtypes of two neuron types: striatal spiny projection neurons, and external globus pallidus neurons. We demonstrated the utility of the covariance matrix adaptation evolutionary strategy by tuning each model type to several sets of experimentally measured membrane potential responses to current injection. Each optimization required ~1 day of simulation time and only 1,600–4,000 evaluations, suggesting that a powerful supercomputer could be used to tune models to large data sets reasonably quickly. Statistical analysis of the resulting parameter sets revealed a small set of parameters that varied between neuron subtypes, indicating that this data-driven modeling approach would be a useful technique for identifying differences between neuron subtypes.

The use of declarative model specification instead of procedural model specification is considered best practice in model development (Gewaltig and Cannon, 2014). A declarative model specification simplifies inspection of the model, and facilitates re-use and extension of the model. The most comprehensive declarative model specification language for multi-compartment, multi-channel models is NeuroML version 2 (Gleeson et al., 2010; Cannon et al., 2014). Its support by both MOOSE and NEURON would simplify exchange of models between simulators. One limitation with NeuroML is that the declarative specification for calcium dynamics is not yet developed; hence the difficulty in using the current NeuroML for our MOOSE models. Nonetheless, implementing a declarative parameter specification with organization and keywords similar to NeuroML will facilitate translation into NeuroML in the near future. A second key feature of our parameter optimization software is to have the optimization wrapped around existing models, similar to some existing optimization algorithms (Friedrich et al., 2014). An advantage of our optimization wrapper is that it keeps the declaration of the parameters and morphology declarative, in contrast to some other approaches (e.g., Brookings et al., 2014; Van Geit et al., 2016). In other words, the parameters for tuning are specified separately from the base model code, both for MOOSE models and for signaling pathway models that are specified and simulated in NeuroRD (https://github.com/neurord/neurord_fit). This approach eliminates the need either to re-specify the model using optimization specific annotations or to insert parameter ranges directly into the base model code.

One limitation of our current optimization software is the inability to adjust half activation and time constants of channel gating for the ionic channels. An initial set of optimizations of the GPe neurons (results not shown) revealed that activation of the hyperpolarization activated cyclic-nucleotide gated (HCN) current in response to hyperpolarizing currents produced a “sag” that was much faster than observed experimentally. To improve this aspect of the fit, the time constant of one of the HCN currents was increased, and the optimizations illustrated all used this slower HCN channel. Given the number of ionic channels activated during action potentials, this hand-tuning approach is not practical for depolarization activated channels. The inability to tune channel characteristics may have contributed to the lower quality fits for the SP neurons. Currently, the software can adjust half activation of one of the channels; thus it will be straight forward to add the capability for all channels. Adding in these parameters should improve the ability to fit the model (Hendrickson et al., 2011b; Brookings et al., 2014; Neymotin et al., 2017), though it would double the number of parameters to tune.

CMA-ES was selected because it has properties which make it appropriate for fitting of complicated and slow-to-simulate models to experimental data: it is robust in the face of local fluctuations of the fitness function, deals well with a high-dimensional and discontinuous fitness landscape, and finally, is frugal with the number of required evaluations, especially compared to other evolutionary algorithms. CMA-ES has been applied to determine protein conformation (Bourquard et al., 2015), and parameters for spiking neuron models (Rossant et al., 2011). A benefit of this algorithm is its fast convergence time, even with large numbers of parameters. Though some parameter optimization algorithms suffer severe slowdowns when the number of parameters is increased, CMA-ES does not suffer from this problem until parameter numbers reach hundreds to thousands (Hansen and Kern, 2004; Hendrickson et al., 2011b; Friedrich et al., 2014; Neymotin et al., 2017). An approach to limit the number of parameters is to perform optimizations in several steps, such as optimizing the passive properties first and spiking activity second (Rumbell et al., 2016), optimizing parameters for proximal conductances to data collected from a neuron with the apical dendrite occluded (Bahl et al., 2012), or using data collected from somatic followed by dendritic recordings (Hay et al., 2011). Though this stepwise approach could facilitate parameter fitting using CMA-ES, avoiding a multi-step approach has the advantage of simplifying the model fitting procedure (conserving the work required from the scientist), and avoids the pitfall where various parameters are strongly correlated and the result of a multi-step fit differs from a single-step fit. Furthermore, fitting to passive properties can underestimate membrane resistance when channels have some activity at resting potential (Keren et al., 2009).

Several studies demonstrate that additional sources of data better constrain the fits. In other words, using measures at two spatial locations (Keren et al., 2009; Hay et al., 2011) or with pinched dendrite (Bahl et al., 2012) better constrains the data. Another data source is calcium dynamics, with simultaneous measures of calcium dynamics and electrical activity (Nevian and Sakmann, 2004; Day et al., 2008; Johenning et al., 2015; Ryu et al., 2017) providing dual contraints. When creating models of calcium dynamics, typically the buffer and pump properties (analogous to channel kinetics) are known (Lee et al., 2000), but pump density and buffer quantity are unknown and need to be adjusted (analogous to channel density). Given the ability of the software to model calcium dynamics, a logical extension would be to optimize to both electrical activity and calcium dynamics measurements. Adding in the calcium dynamics optimization includes reading in calcium imaging data and adapting the fitness function to calcium.

One of the difficult aspects of optimization is designing a fitness function that captures the perceived similarity between simulated and measured voltage traces (or calcium dynamics). One approach is to perform a point-by-point match to the voltage trace. This measure is problematic for neuron activity due to the narrow time window of spikes. A clever approach to avoid this problem has been implemented (Abarbanel et al., 2009; Brookings et al., 2014) and avoids sensitivity to the fitness functions selected. Unfortunately, the custom code to implement this approach is not written for an existing simulator; however, it would be interesting to incorporate that approach into a fitness function for use with MOOSE. A second approach is to use features of the data, such as spike time, width, height, AHP shape as well as non-spiking features. The large number of features can be combined into a single feature, used individually in multi-objective optimization (Druckmann et al., 2007; Rumbell et al., 2016; Neymotin et al., 2017), or combined into one (or a few) combined features (Keren et al., 2009; Rumbell et al., 2016). One rationale for performing a multi-objective optimization is that an overall best match may not be possible; instead a multi-objective optimization provides a set of optimal solutions that represent the best trade-offs between conflicting objectives. Using multi-objective optimization also avoids the process of assigning weights to features, which by definition are to some extent arbitrary. Nevertheless, after obtaining the set of optimal solutions from a multi-objective optimization, finding one solution that achieves a good fit of all features may be difficult. We opted to combine multiple objectives (features) into a single fitness value, effectively preferring solutions that performed moderately well on all measures to those which were optimized toward some specific subset of features. Early explorations using multi-objective optimization yielded models that indeed fitted some features well, but at the same time were divergent enough in other characteristics that if observed experimentally, such neurons would be classified as a different type. For real neurons, natural variability exists between inviduals of the same type, and also between repeated measurements, yet the defining features are common to all neurons of a certain type. We feel that fitting very precisely to *some* characteristics of an invidual experimental measurement is less useful than fitting *all* features approximately.

An important concept utilized by multi-objective optimization is weighting the various feature fitnesses by variance across the data, under the assumption that more variable features should be less constrained. Weighting by variance (i.e., dividing by the standard deviation) also removes dimensionality from the data (e.g., dividing a difference of 10 mV by a standard deviation of 1 mV yields the dimensionless number of 10). This procedure allows fitness values of features with both small values (e.g., spike width measured in seconds) and large values (e.g., spike height measured in mV) to contribute meaningfully to the total fitness. Whereas the variance for the spike characteristics can be calculated within a neuron, a better variance estimate requires recordings of multiple trials or multiple neurons (Hendrickson et al., 2011b). Our algorithm removes dimensionality from the feature fitnesses by dividing the difference between data and simulations by the mean. The software also allows a weight to be specified, which could be (the inverse of) the variance between neurons. Clearly, another improvement to the software would be to add a module to calculate and use the variance between neurons either when current injection protocols are repeated several times or when multiple data sets are available.

A major concern with using parameter optimization to identify differences between neuron types is that unique parameter sets do not exist (Golowasch et al., 2002; Prinz et al., 2003b; Olypher and Calabrese, 2007; Hay et al., 2011). Instead there are multiple valid parameter sets with parameter co-variation, which hinders the ability to classify neurons based on these conductance parameters. Though CMA-ES takes into account these correlations during the optimization, CMA-ES does not find all parameter sets, since it continually seeks a (single) global minimum. In principal, CMA-ES could be initiated from different points in parameter space to find multiple local minima. Even with a single run of CMA-ES per neuron recording, analysis of the best parameter sets revealed several correlations between conductances when all models of a neuron subtype were considered. The most common correlations were compensatory, with increases in inward currents correlated with increases in outward currents, or increases in one type of potassium current correlated with a decrease in a different type of potassium current. Interestingly, most correlations were not observed for a single neuron, but were observed across the set of neurons, suggesting that differences in that set of conductances may represent natural variation within neuron subtypes (Taylor et al., 2009).

Optimization of several exemplars allowed us to evaluate differences between neuron subtypes. Experimentally, low frequency firing neurons of the globus pallidus, such as the arkypallidal neurons, show a slight increase of firing rate when the BK channel is blocked (Abrahao et al., 2017). In addition, ethanol (which directly targets the BK channel) does not affect the firing rate of high frequency firing, prototypical neurons of the globus pallidus; but does decrease the firing rate of low frequency GPe neurons by increasing the open probability of BK channels (Abrahao et al., 2017). These experimental data suggest that arkypallidal and prototypical neurons have different conductance of BK channels, as suggested by statistical analysis of the arkyN and protoN parameters. ArkyN and protoN neuron models also differed in transient potassium conductance, which has been reported experimentally (Hernández et al., 2015). The HCN channel also has been characterized in arkypallidal and prototypical neurons, with one report of a difference (Hernández et al., 2015) and one report of no difference (Mastro et al., 2014). Our observation of no difference in HCN currents between subtypes is consistent with the latter publication, but it is not inconsistent with the data from the former which shows that strong hyperpolarization is required to observe the greater sag ratio of PV− vs. PV+ neurons.

The optimization also reported that ArkyN had higher capacitance than ProtoN, a difference that is not supported experimentally. One possible cause of this discrepancy is the use of the same morphology for all GPe optimizations, since using a different morphology changes the fitted passive parameters (Holmes et al., 2006). The neurons from which electrophysiology data were obtained have not been reconstructed, precluding using the morphology that matches the data. In addition, the optimization may have (incorrectly) increased the ArkyN capacitance to produce shallow AHPs, to compensate for the present inability to adjust time constants and half activation values of the potassium currents. Note that the classification of arkypallidal vs. prototypical neurons is based on firing characteristics, with recent attempts to identify these neurons based on biochemical markers. There is broad agreement than PV+ neurons are prototypical, but PV− neurons can be prototypical cells, expressing Lhx6 (Mastro et al., 2014), or arkypallidal cells, expressing Npas1+ or FoxP2+ (Dodson et al., 2015; Hernández et al., 2015; Glajch et al., 2016). In fact, there are both similarities (HCN conductance) and differences (transient potassium current) between the Npas1+ and Lhx6+ neurons. Future parameter optimization of morphlogically reconstructed neurons exhibiting these different markers may better determine ionic conductance differences among all these neuron types. Ideally, easy-to-use, automatic approaches for identifying neuron channel parameters may facilitate experiments used to characterize such differences.

## Author contributions

ZJ-S: modeling and optimization software development, manuscript preparation; JJ-S: modeling software development, manuscript preparation; KA: GPe experiments, manuscript preparation; DL: GPe experiments, manuscript preparation; KB: SP experiments, modeling software development, model simulation and analysis, manuscript preparation.

### Conflict of interest statement

The authors declare that the research was conducted in the absence of any commercial or financial relationships that could be construed as a potential conflict of interest.
