# Supplementary figures and images for: Parameter Optimization Using Covariance Matrix Adaptation—Evolutionary Strategy (CMA-ES), an Approach to Investigate Differences in Channel Properties Between Neuron Subtypes
Source: Front Neuroinform. 2018 Jul 31;12:47. doi: 10.3389/fninf.2018.00047 (PMC6079282; doi:10.3389/fninf.2018.00047)

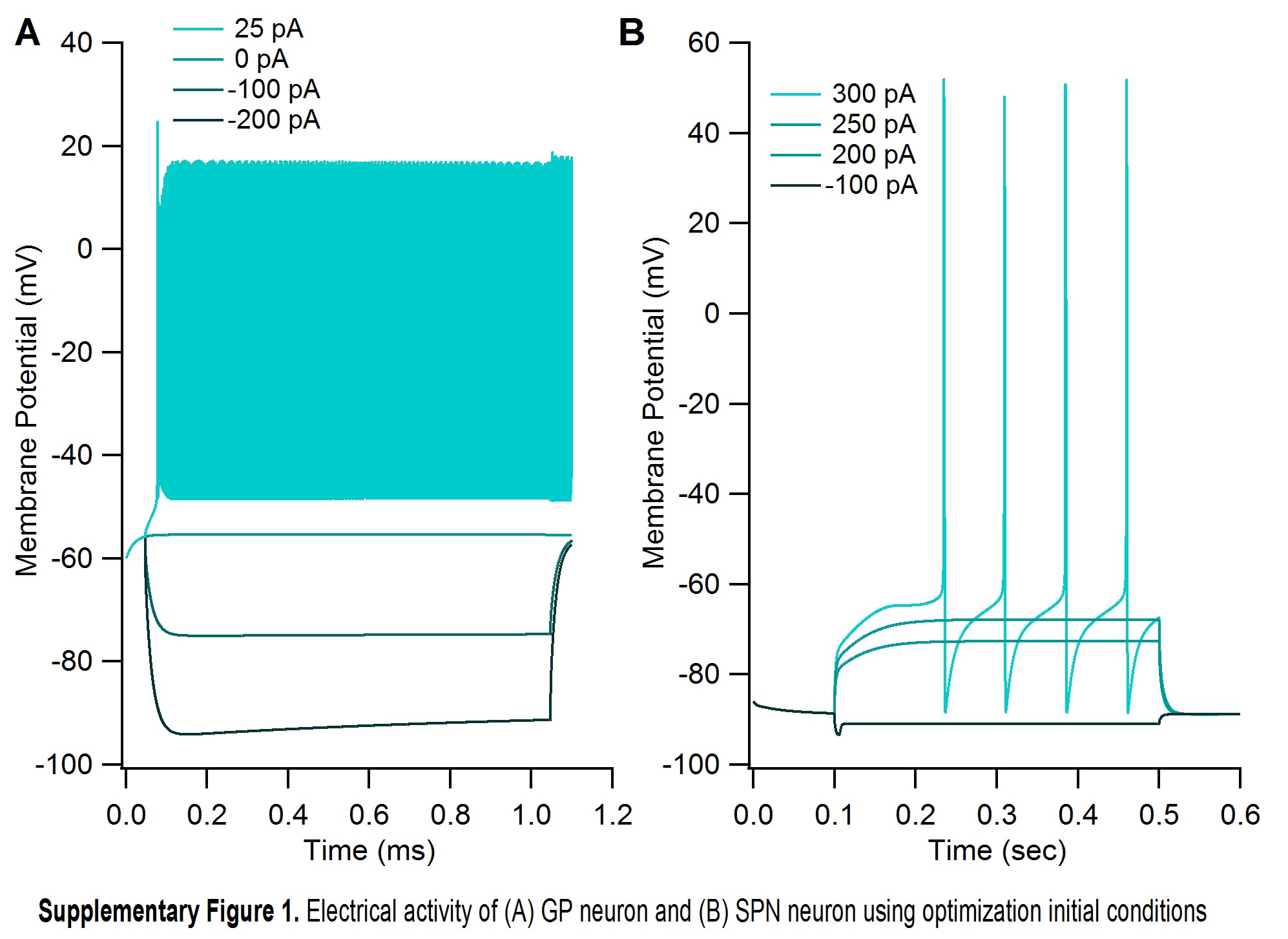

Supplement: Supplementary file 1 [file Image_1.JPEG]
